# Uuse of a dashboard based on rapid viral tests results for predicting and managing clinical services

**DOI:** 10.1186/1753-6561-5-S6-P327

**Published:** 2011-06-29

**Authors:** CHS Lam, PTY Ching, WH Seto

**Affiliations:** 1 Hospital Authority, Hong Kong, Hong Kong, China

## Introduction / objectives

Viral culture results (VCT) correlates well with the intensity of clinical services but they are not available real time. Rapid viral tests results (RVT) are available on the same day and can be used for real time predictions. This study reports the use of RVT for such prediction for a period of 145 weeks in Hong Kong (HK). Two acute hospitals successfully use them to manage the winter Influenza surge of 2009.

## Methods

Viral cultures are provided by the government Public Health Laboratory Centre (PHLC). The public hospitals accounting for 91% of hospital beds in HK provide RVT’s by immunofluorence and is done in five hospital laboratories. Since May 09, PCR is also available for the diagnosis of nH1N1. Admissions data is obtained from the public hospital computer system. Two hospitals when alerted by the summary of the RVT results initiated a program to manage the influenza surge by rapid discharges, doctor’s visits to old age homes to reduce hospital admissions and doctors rights of admission to any ward with available beds.

## Results

The RVT results (% positive) correlates closely with the number of weekly positive cultures of the PHLC for the 145 weeks (r = 0.85; p < 0.001). There is significant correlation of RVT for Influenza and admissions for children (Influenza A: r = 0.94, p < 0.001) and the elderly (Influenza A: r = 0.77, p < 0.001). During the winter surge, % of admissions in the 15 acute hospitals is statistically similar with a mean of 12% each. The two acute hospitals with surge management have an occupancy rate of 81% and 84% while the others have a mean of 105% (p<0.001, chisquare).

## Conclusion

The RVT for Influenza is a good predictor of clinical services intensity and can help in managing the surge in clinical services.

## Disclosure of interest

None declared.

